# Sensitivity Enhanced Plasmonic Biosensor Using Bi_2_Se_3_-Graphene Heterostructures: A Theoretical Analysis

**DOI:** 10.3390/nano12224078

**Published:** 2022-11-19

**Authors:** Fusheng Du, Kai Zheng, Shuwen Zeng, Yufeng Yuan

**Affiliations:** 1 School of Electronic Engineering and Intelligentization, Dongguan University of Technology, Dongguan 523808, China; 2 School of Information and Optoelectronic Science and Engineering, South China Normal University, Guangzhou 510631, China; 3 Shenzhen Key Laboratory of Photonics and Biophotonics, Key Laboratory of Optoelectronic Devices and Systems of Ministry of Education and Guangdong Province, College of Physics and Optoelectronic Engineering, Shenzhen University, Shenzhen 518060, China; 4 School of Civil Aviation, Northwestern Polytechnical University, Xi’an 710072, China; 5 Light, Nanomaterials & Nanotechnologies (L2n), CNRS-ERL 7004, Université de Technologie de Troyes, 10000 Troyes, France

**Keywords:** plasmonic biosensor, Bi_2_Se_3_-Graphene heterostructures, differential GH shift, ultrasensitive biosensing

## Abstract

This study provided a theoretical insight for designing novel plasmonic biosensors using bismuth selenide (Bi_2_Se_3_)-Graphene heterostructures. It was a van der Waals (vdWs) stacked configuration composed of gold (Au) film, few quintuple layer (QL) Bi_2_Se_3_ and few-layered graphene. In particular, the proposed biosensor was created by Goos-Hänchen (GH) shift rather than phase, resulting in a more sensitive biosensing response. Under the excitation of 632.8 nm, significant sensitivity enhancement performance was obtained via varying the thickness of Bi_2_Se_3_-Graphene heterostructures. The best configuration was 32 nm Au film−2-QL Bi_2_Se_3_-3-layer graphene, generating the largest GH shift, as high as −1.0202 × 10^4^ µm. Moreover, the highest detection sensitivity was determined to be 8.5017 × 10^6^ µm/RIU, responding to a tiny refractive index (RI) change of 0.0012 RIU (RIU, refractive index unit). More importantly, our proposed biosensor has shown a theoretical feasibility of monitoring virus samples. For example, there was an efficient linear detection range for severe acute respiratory syndrome coronavirus 2 (SARS-CoV−2, 0~13.44 nanomole (nM)) and its Spike (S) glycoprotein (0~59.74 nM), respectively. It is expected that our proposed plasmonic biosensor has a potential application in performing sensitive detection of SARS-CoV−2.

## 1. Introduction

Surface plasmon resonance (SPR) biosensors are a class of important optoelectronic devices widely employed in the biomedicine science field. To date, significant breakthroughs in plasmonic biosensing are focused on the following two studies: (i) novel plasmonic materials and (ii) optimization of biosensing process [1]. For plasmonic materials, the common plasmonic substrates are metallic conductor films, such as gold, silver and aluminum. To obtain biosensing performance, four signal-modulation methods, including angle-, amplitude-, wavelength- and phase-modulation, are widely employed [2]. However, it is unfeasible to achieve noteworthy development using standard biosensing procedure. Conversely, adopting novel plasmonic materials could provide extraordinary exploration. For example, graphene, a well-known member of two-dimensional (2D) materials family, firstly paved a way to design novel SPR biosensing devices using 2D vdWs material [3]. Afterwards, other 2D layered semiconductor materials, such as transitional metal dichalcogenides (TMDCs), [4,5] phosphorene [6,7], antimonene [8] and tellurene [9], have been considered as promising plasmonic materials for creating novel plasmonic biosensors. It is generally accepted that common solid plasmonic materials can be categorized into three classes: conductor, semiconductor and insulator. In the 2D material family, graphene is an excellent conductor at room temperature. Both TMDCs and phosphorene are classical representatives of semiconductor materials. Hexagonal boron nitride (h-BN), known as white graphene, belongs to insulators. However, topological insulators (TIs) do not belong to any of the above classes. Recently, TIs are a new class of promising electronic materials, which have fascinating helical metal surface states and bulk-insulating bandgaps [10]. Interestingly, the metallic surface states cause electrons to accumulate and transfer at the surface. Under the illumination of incident photons, regular electron transfer can form surface plasmon polaritons (SPPs) wave, and SPR effect can be efficiently excited by optimal wave vector matching condition. Owing to their exotic electronic features, TIs have shown great potential for next-generation photonic biosensors. It is worth noting that, due to strong spin-orbit coupling, the surface electrons in TIs can obey a massless Dirac equation, forming a Dirac cone system, which was observed in only graphene atomic monolayer [11]. Then, Dirac charge carriers could form a current and freely move parallel to the topological surface [12]. In early 2013, Dirac plasmons was successfully observed [13]. Unlike noble metal and semiconductor thin films, Bi_2_Se_3_ film has unusual Dirac plasmons. Thus, Bi_2_Se_3_ film shows an enhanced sensitivity for RI variation [14]. In addition, TIs films usually have a broad photon absorption [15]. It is possible that the broad plasmon modes are able to interact with other phonon modes, producing typical Fano-resonance narrow lines. Therefore, TIs are promising candidates to explore exotic plasmonic biosensors.

It is well-known that TIs family has several prototypical members, which can be described as M_2_X_3_. Here, M stands for the element Bi or Sb, and X denotes the element Te or Se. Bi_2_Se_3_ is the most common 2D topological insulator material, whose crystal lattice consists of quintuple layers (QL) orderly arranged by a Se–Bi–Se–Bi–Se unit [16,17]. In addition, the QLs can be stacked on each other through weak vdWs interaction. Moreover, Bi_2_Se_3_ has shown many unique features, such as a large bandgap of ~0.3 eV [18], well-defined Dirac plasmons [10], high photothermal-conversion ability [19,20] and electrochemical catalytic capacity [21]. It is possible that, stacking novel vdWs heterostructures using various 2D materials can produce unprecedented physical and electric features. More recently, topological insulator/graphene heterostructures are proposed to be an efficient solution to significantly enhance the photon response and carrier mobility. For example, Chae et al. reported that by introducing 10-layer graphene, 10 QL Bi_2_Se_3_ films exhibited the maximum photon response [22]. At room temperature, the carrier mobility of Bi_2_Se_3_ films with a thickness less than 22 nm is 50~200 cm^2^V^−1^s^−1^ [23]. However, the carrier mobility could be significantly increased by integrating with graphene. At a low temperature, the carrier mobility of 400 nm Bi_2_Se_3_/Graphene heterostructures was observed as high as 5000~6000 cm^2^V^−1^s^−1^ [24,25]. Moreover, the carrier mobility in Bi_2_Se_3_/graphene heterostructures at room temperature can be optimized to be 3400 cm^2^V^−1^s^−1^ [23]. To the best of our knowledge, there were few observations on creating plasmonic biosensors using topological insulator materials.

Inspired by these observations, we theoretically proposed a novel plasmonic biosensor using vdWs Bi_2_Se_3_-Graphene heterostructures to linearly detect SARS-CoV−2 and its S glycoprotein. Rather than phase-modulation, our proposed SPR biosensor was designed by GH shift, showing a higher biosensing performance. Both photon absorption and energy dissipation were optimized to be a balance state by varying thickness of Bi_2_Se_3_/Graphene heterostructures. The optimal configuration: 32 nm Au film−2-QL Bi_2_Se_3_–3-layer graphene could provide the largest GH shift up to −1.0202 × 10^4^ µm. For a tiny RI variation of 0.0012 RIU, the highest detection sensitivity of 8.5017 × 10^6^ µm/RIU can be achieved. Moreover, the proposed biosensor could be employed to simulate the adsorption behavior of SARS-CoV−2 and its S glycoprotein. A good linear response interval for monitoring SARS-CoV−2 was from 0 to13.44 nM. For S glycoprotein, a theoretical linear detection interval was from 0 to 59.74 nM. In all, the optimal biosensor is a potential candidate in quantitatively monitoring SARS-CoV−2 and other infectious viruses in practical clinical applications.

## 2. Methodology

**Figure 1:**
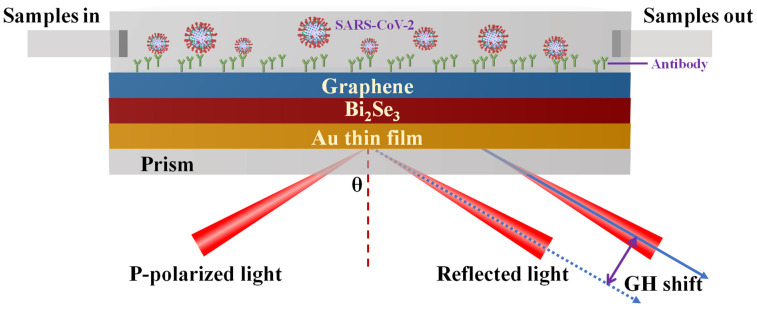
Schematic diagram of TI-enhanced plasmonic biosensor.

The theoretically proposed SPR biosensor consists of an SF11 prism, an Au thin film, few layered Bi_2_Se_3_ interlayer and graphene overlayer, as shown in Figure 1. Firstly, the plasmonic gold film was efficiently integrated with a SF11 prism. It is worth noting that plasmonic metal materials usually suffer from strong energy dissipation because of inter-band electronic transitions [26]. To further enhance detection sensitivity, it is necessary to supplement low-loss plasmonic materials onto gold film. Considering the vdWs stacked Bi_2_Se_3_/Graphene heterostructures having high carrier mobility, it was introduced to work as an important plasmonic layer, significantly developing the energy dissipation. Then, Bi_2_Se_3_ interlayer was vdWs stacked onto the top of plasmonic gold film. After this, few layered graphene overlayer was further stacked onto Bi_2_Se_3_ interlayer to form Bi_2_Se_3_/Graphene heterostructures. The topmost layer was a sealed cell containing work solutions. It is worth noting that the work solutions consisting of 10 mM HEPES (4-(2-hydroxyethyl)−1-piperazineethanesulfonic acid) and 120 mM NaCl (sodium chloride) was utilized to contain SARS-CoV−2 and its S glycoprotein in this simulation. To really calculate the binding interaction between SARS-CoV−2 and sensing interface of Bi_2_Se_3_/Graphene heterostructures, the work buffer consisting of 10 mM HEPES and 120 mM NaCl solutions was injected from left entrance. Once the target analytes in running buffer were bound to the sensing interface, the change in local RI would be different. Generally, the tiny variation in RI could be readout by measuring the change in intensity of reflected light, SPR angle and differential phase. In this work, p-polarized incident light having a wavelength at 632.8 nm was applied to resonate with SPPs wave in Au film/Bi_2_Se_3_/Graphene heterostructures. Unlike previous SPR biosensors created by phase modulation, our proposed biosensor was designed by measuring GH shift. It is well-known that GH shift is an interesting displacement behavior of reflected light relative to geometric reflection theory, because practical incident lights generated by lasers are not ideal monochromatic electromagnetic plane waves [27]. Moreover, GH shift can be explained by stationary phase theory [28], indicating that GH shift was highly related with phase transition of reflected light. Specifically, the GH shift can be obtained by calculating the first derivative of reflected light versus incident angle (Equation (12)). However, the actual GH shifts generated by low RI dielectric interface were only 5~10 μm [29,30], which was difficult for experimental measurement. Fortunately, GH shifts could be hugely enhanced using SPR technology [31,32]. Under the strongest SPR excitation, phase of reflected light usually experiences a steep jump and GH shift is the first-order derivative of phase. Then, GH shift was supposed to be more sensitive than phase transition under the strongest SPR effects. Therefore, GH shift could work as an important indicator for evaluating biosensing performance for our proposed biosensor. However, compared with other measurement models, including angle-, amplitude-, wavelength- and phase-modulation, large GH shifts usually suffer from a narrow interval for tuning incident angle.

Both photon absorption and energy dissipation in our proposed configuration highly depend on optical dielectric constant of each layer. Prior to biosensing simulation, the dielectric constant of each layer needs to be determined. Under the excitation light of 632.8 nm, the refractive indices of SF11 prism layer, Au film layer, Bi_2_Se_3_ nanosheets and graphene overlayer is 1.7786 [33]. 0.1838 + 3.4313i [7]. 4.2923 + 1.7922i [34] and 3.000 + 1.1487i [7], respectively. In addition, the thickness of 1-QL Bi_2_Se_3_ and monolayer graphene is 0.9725 nm [34] and 0.34 nm [35], respectively. Finally, the refractive index of running buffer containing 10 mM HEPES and 120 mM NaCl solution can be calculated by Equation (1):

(1) y=0.00004x+1.3341

where y stands for the calculated RI of running buffer and x is the concentration of HEPES solution (mM). In this simulation, 10 mM HEPES solution was added into 120 mM NaCl solution and the refractive index of the running buffer (n_C_) can be determined to be 1.3345 [9]. When S glycoproteins based on SARS-CoV−2 was captured antibody sites on graphene layer, the refractive index of sensing interface based on Bi_2_Se_3_/Graphene configuration was assumed to be proportional to the concentration of S glycoprotein, which can be calculated by Equation (2):

(2) $$n_{A}-n_{C}=\left(dn/dc\right)c_{A}$$

where *n_A_* is the RI due to the adsorption interaction of S glycoprotein and *n_C_* is the RI of running buffer. *c_A_* is the concentration of S glycoprotein solution. In addition, the parameter (*dn*⁄*dc*) denotes the increment of refractive index and the value of *dn*⁄*dc* is usually ~0.186 cm^3^•g^−1^ for studying protein-protein binding interactions [36]. For a fixed RI variation in sensing interface, the concentration (*c_A_*) of S glycoproteins can be calculated according to the binding parameter (*dn⁄dc).*

In addition to SPR reflectivity, change in incident angle and differential phase, change in GH shift generated by plasmonic enhancement in our proposed SPR configuration was systematically simulated using transfer matrix method (TMM) and Fresnel equations in a N-layer stacked model. Theoretically, each layer was supposed to parallel in Z-direction and each layer was considered to be isotropic and non-magnetic. It was assumed that the number of antibody sites on graphene layers was much more than that of S protein. The adsorption sites on graphene overlayer were S protein antibody, which can be employed for specifically capture SARS-CoV−2 using antibody-antigen interactions. Generally, the electromagnetic fields at the first boundary along the tangential direction was supposed to be Z_1_ = 0, and the relationship between the last boundary ZN−1 and first boundary Z1 could be given as:

(3) $$\left[\begin{array}{c} U_{1}\\ V_{1} \end{array}\right]=M\;\left[\begin{array}{c} U_{N-1}\\ V_{N-1} \end{array}\right]$$

where U and V denote the tangential components of electric fields and magnetic field at the boundary, respectively.

Here, M is a transfer matrix, which can be obtained by the following equation for p-polarized light:

(4) $$M=\overset{N-1}{\underset{k=2}{\prod }}M_{k}=\left[\begin{array}{cc} M_{11} & M_{12}\\ M_{21} & M_{22} \end{array}\right]\;$$

where M_k_ could be described as:

(5) $$M_{k}=\left[\begin{array}{cc} \cos\beta_{k}\; & \left(-i\sin\beta_{k}\right)/q_{k}\\ -iq_{k}\sin\beta_{k} & \cos\beta_{k} \end{array}\right]\;$$

where *β_k_* and *q_k_* can be determined using the following two Equations (6) and (7):

(6) $$\beta_{k}=\frac{2\pi d_{k}}{\lambda }{\left(\varepsilon_{k}-n_{1}^{2}\sin^{2}\theta_{1}\right)}^{\frac{1}{2}}$$

(7) $$q_{k}=\frac{{\left(\varepsilon_{k}-n_{1}^{2}\sin^{2}\theta_{1}\right)}^{\frac{1}{2}}}{\varepsilon_{k}}$$

where k is the k-th stacking layer and *d_k_* stands for the thickness of the k-th layer. Additionally, *ε_k_* represents the dielectric constant of the k-th layer. In Equation (7), *θ_1_* and *n_1_* are the incident angle and RI of the first stacking layer, respectively.

For s-polarized light, these relationships described above are still practicable. However, *q_k_* was described by Equation (8):

(8) $$q_{k}={\left(\varepsilon_{k}-n_{1}^{2}\sin^{2}\theta_{1}\right)}^{\frac{1}{2}}$$

Prior to calculating GH shift, the reflectivity (*R_p_*) in our proposed configuration can be obtained using Equation (9):

(9) $$R_{p}=r_{p}^{2}={\left|\frac{\left(M_{11}+M_{12}q_{N}\right)q_{1}-\left(M_{21}+M_{22}q_{N}\right)}{\left(M_{11}+M_{12}q_{N}\right)q_{1}+\left(M_{21}+M_{22}q_{N}\right)}\right|}^{2}$$

Next, the p-polarized of reflected light phase *φ_p_* can be calculated by the following equation:

(10) $$\varphi_{p}=\arg\left(r_{p}\right)$$

Therefore, the differential phase Δ*φ_d_* can be calculated by Equation (11):

(11) $$\Delta \varphi_{d}=\left|\varphi_{p}-\varphi_{s}\right|\;$$

where *φ_s_* stands for the phase of s-polarized light.

According to the stationary phase theory, the GH shift (*GHS*) can be calculated by Equation (12):

(12) $$GHS=-\frac{\lambda }{2\pi }\frac{d\varphi }{d\theta }\;$$

For p-polarized light, the GH shifts produced by metal films are negative. However, the GH shift excited by s-polarized light in metal films are usually positive [37]. Moreover, the value of GH shift from p-polarized light is much larger than s-polarized light. Similar to the differential phase, the differential GH shift (Δ*GHS_d_*) between p- and s-polarized light can be calculated as follows:

(13) $$\Delta GHS_{d}=\left|GHS_{p}-GHS_{s}\right|$$

To evaluate the biosensing performance of our proposed configuration, two parameters, such as phase sensitivity (*S_φ_*) and GH shift sensitivity (S_GHS_), were introduced. Both of them can be determined as follows:

(14) $$S_{\varphi }=\frac{\Delta \varphi_{d}}{\Delta n_{bio}}\;$$

(15) $$S_{GH}=\frac{\Delta GHS_{d}}{\Delta n_{bio}}\;$$

where Δ*n_bio_* denotes a tiny variation of RI in sensing interface of Bi*_2_*Se_3_/graphene heterostructures due to strong adsorption binding interaction.

## 3. Results and Discussion

**Figure 2:**
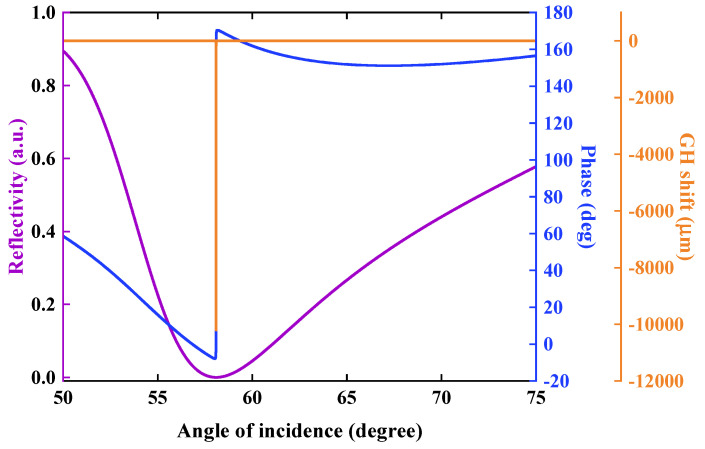
Reflectivity (purple), phase (blue) and GH shift (orange) with respect to the angle of incidence. The thickness of Au thin film is 32 nm. The number of Bi_2_Se_3_ and graphene layer numbers is 2 and 3, respectively. The wavelength of incident light is 632.8 nm.

Under the illumination of 632.8 nm, the optimal reflectivity, phase and GH shift was obtained, as shown in Figure 2. It can be found that when the incident angle is located at 58.0864°, the stacked configuration: 32 nm Au film−2-QL Bi_2_Se_3_–3-layer graphene can produce an ultralow reflectivity (purple curve, Figure 2) of 5.4402 × 10^−9^, approaching zero. It indicates that almost 100% of photons were absorbed and converted into SPR energy. Meanwhile, the phase produces a sharp transition at the same incident angle (blue curve, Figure 2), perfectly corresponding to the minimum SPR reflectivity. More importantly, the GH shift showed a sharper transition strength than phase at the point of SPR angle. There was a significant enhanced negative GH shift (orange curve, Figure 2) in Au film/Bi_2_Se_3_/graphene heterostructures as high as −1.0202 × 10^4^ µm. These observations suggest that a large GH shift is a useful indicator for evaluating biosensing performance.

**Figure 3:**
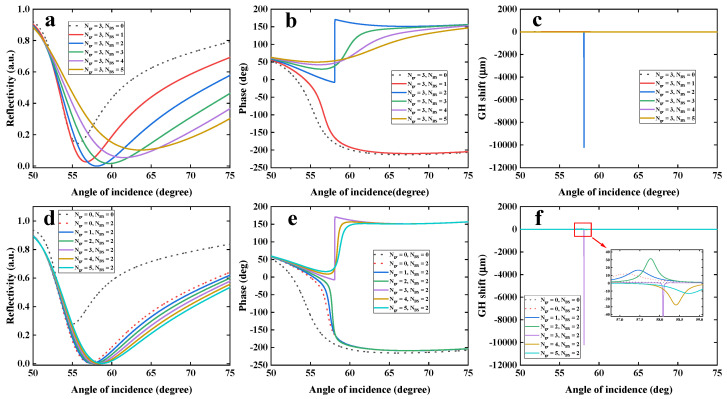
Variation in reflectivity (**a**), phase (**b**) and GH shift (**c**) with respect to angle of incidence by varying the number of Bi_2_Se_3_ QLs (0–5 layers). The graphene is fixed to be 2 QLs. The Au thin film thickness is 32 nm and the wavelength of incident light is 632.8 nm. Variation in reflectivity (**d**), phase (**e**) and GH shift (**f**) with respect to angle of incidence by varying the number of graphene overlayer (0–5 layers). The number of Bi_2_Se_3_ QLs is fixed to be 2. Au thin film thickness is 32 nm and the wavelength of incident light is 632.8 nm. Abbreviation: BS in inset stands for Bi_2_Se_3_.

To further verify sensitivity enhancement ability of our proposed configuration, the GH shift as well as reflectivity, incident angle and phase, were systematically examined by tuning the stacking thickness of Bi_2_Se_3_/Graphene heterostructures (Bi_2_Se_3_, 0–5 layers; graphene, 0–5 layers), as shown in Figure 3. In the absence of Bi_2_Se_3_/graphene heterostructures, the plasmonic Au film (32 nm) has a reflectivity of 0.2786 under the illumination of 632.8 nm (black dotted curve, Figure 3a). It means that, single Au film suffers from large energy dissipation. However, the dilemma can be significantly improved by introducing low-loss Bi_2_Se_3_/Graphene heterostructures. When 2-QLs Bi_2_Se_3_ film was stacked onto 32 nm Au film, the reflectivity of Au film-Bi_2_Se_3_ heterostructures could be decreased to 0.0065 (blue curve, Figure 3a). Moreover, as 3-layer graphene was stacked, the SPR reflectivity (blue curve, Figure 3d) of gold film/Bi_2_Se_3_/graphene heterostructures could be lowered to 5.4402 × 10^−9^. In addition, there were obvious red-shifts in incident angle, due to the addition of Bi_2_Se_3_/Graphene heterostructures. Interestingly, the obtained reflectivity of 5.4402 × 10^−9^ corresponds to the optimal incident angle at 58.0864°. Meanwhile, phase from reflected light also experienced a Heaviside step-like transition, as shown in Figure 3b–e. Moreover, it showed the steepest phase singularity as the plasmonic configuration biosensor was stacked as 32 nm Au film−2-QL Bi_2_Se_3_-3-layer graphene. At the phase singularity, the GH shift can be obtained by calculating the derivative of phase with respect to incident angle. It can be found that the largest GH shift of −1.0202 × 10^4^ µm is generated by 32 nm Au film-2-QL Bi_2_Se_3_-3-layer graphene, as shown in Figure 3f. Interestingly, with increasing graphene layers, the value of GH shift changes from positive to negative, which is synchronized with phase transition. Figure 3f showed that as the number of Bi_2_Se_3_ QLs is from 0 to 5, the obtained GH shift is 5.4312, 7.3689, −1.0202 × 10^4^, −3.9267, −1.5591 and −0.8831 µm, respectively. However, Figure 3f also showed that a tunable interval of incident angle for obtaining multiple large differential GH shift is narrow. The reason is that, at the dip of minimum SPR reflectivity, both phase and GH shift of reflected light usually experience significant transition behavior. Moreover, GH shift was obtained by derivating phase with respect to incident angle (Equation (12)). Thus, the GH shift shows a sharper transition strength than phase. The narrow interval of incident angle seems that it will be difficult for performing practical measurement. According to phase-modulation theory, once an optimal incident angle is fixed, both largest differential phase and GH shift will be determined. To perform practical GH measurements, it needs to fix at an optimal incident angle rather than vary incident angle. 

**Figure 4:**
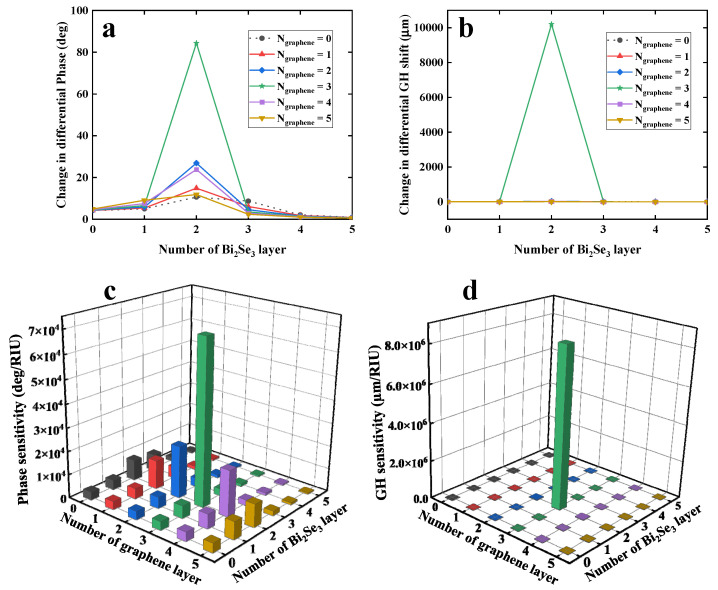
Change in differential phase (**a**) and GH shift (**b**) obtained by tuning the number of Bi_2_Se_3_ QLs (0–5) and graphene layers (0–5) for a defined RI variation of 0.0012 RIU. Obtained phase (**c**) and GH shift (**d**) detection sensitivity obtained by changing the number of Bi_2_Se_3_ QLs (0–5) and graphene layers (0–5).

To study the biosensing ability of our proposed biosensor, both differential phase (Δ*φ_d_*) and GH shift (Δ*GHS_d_*) response for a defined RIU variation (Δ*n_bio_* = 0.0012) were plotted, as shown in Figure 4a,b. The largest differential phase (green curve, Figure 4a) is 84.3891°, generated from the configuration of 32 nm Au film/2QL-Bi_2_Se_3_/3-layer graphene. Figure 4b showed the change in differential GH shift (Δ*GHS_d_*) by changing the number of Bi_2_Se_3_ and graphene layers. The configuration of 32 nm Au film-Bi_2_Se_3_ (2 QL)-graphene (3 layer) provides the highest differential GH shift of 1.0202 × 10^4^ µm (green curve), which is almost 324 times larger than the second highest differential GH shift of 31.466 µm (blue curve) generated by the configuration of 32 nm Au film/2-QL Bi_2_Se_3_/2-layer graphene. When the number of Bi2Se3 QLs is 2, the phase detection sensitivity obtained by varying the number of graphene overlayer are almost on the same order of magnitude of 10^4^°/RIU, as shown in Figure 4c. However, the difference in differential phase can be hugely amplified by further calculating derivative of phase. Thus, the obtained GH shift detection sensitivity shows a larger difference than phase detection sensitivity, as shown in Figure 4d. Thus, the change in GH shift was more significant compared to phase modulation.

In addition, we also studied the changes in incident angle (Δ*θ_SPR_*) for a defined RIU variation (Δ*n_bio_* = 0.0012), as shown in Figure S1. There was only a response of 0.0126° for red-shift in SPR angle. However, such tiny red-shifts in SPR angle cannot be distinguished by an optical detector. Conversely, both differential phase and GH shift can be employed to monitor the RI variation of sensing interface. Afterwards, the obtained largest phase sensitivity was 7.0324 × 10^4^ degree/RIU, as shown in Figure 4c. In contrast, Figure 4d showed the largest GH shift sensitivity could reach the value of 8.5017 × 10^6^ µm/RIU. It is generally agreed that such a large GH shift can be easily detected by common optical devices. Compared with other reported GH shift-based plasmonic configurations (Table 1), our proposed biosensor exhibited higher detection sensitivity. In addition, considering that few-layered Bi_2_Se_3_ nanosheets have the advantages of simple fabrication and low cost [38], our proposed biosensor will be inexpensive and easily fabricated.

**Figure 5:**
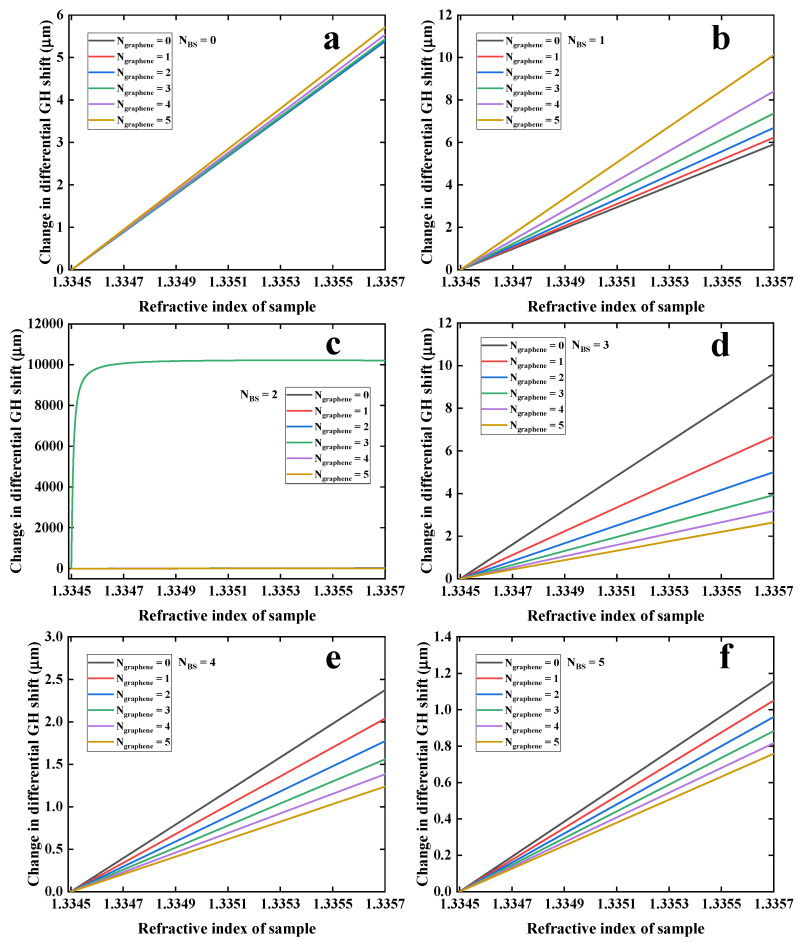
Change in differential GH shift with respect to change in RI of sensing interface by varying the number of graphene (0–5) and Bi_2_Se_3_ QLs: (**a**) 0 layer, (**b**) 1 layer, (**c**) 2 layers, (**d**) 3 layers, (**e**) 4 layers and (**f**) 5 layers. Abbreviation: BS in inset stands for Bi_2_Se_3_.

Next, the detailed changes in differential GH shift for the RI from 1.3345 to 1.3357 RIU by tuning the number of Bi_2_Se_3_ QLs (0–5) and graphene overlayers (0–5) were studied, as shown in Figure 5. When the number of Bi_2_Se_3_ layers was less than 2, there was a good linear response for change in differential GH shift by changing the RI of sensing interface. Obviously, the obtained GH shift was relative weak, indicating that there was no significant SPR enhancement effects, as shown in Figure 5a,b. However, when the number of Bi_2_Se_3_ layers is 2, the addition of 3-layer graphene can precipitously produce an ultrasensitive response, as shown in Figure 5c. When the number of Bi_2_Se_3_ layers was larger than 2, the addition of graphene overlayer could not provide a positive response because of excessive energy loss. It can be concluded that the configuration of 32 nm Au film coated with two-QL Bi_2_Se_3_ and three-layer graphene has the strongest GH shift response, showing great promise for performing ultrasensitive biosensing for a tiny RI variation.

**Figure 6:**
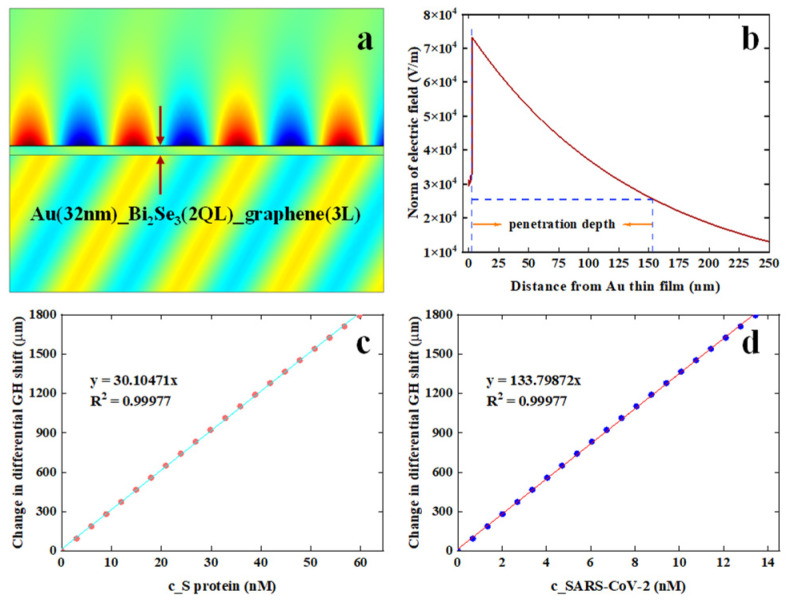
(**a**) The distribution of highly enhanced electric field approaching to Bi_2_Se_3_/graphene sensing interface. (**b**) Decay curve of highly enhanced electric field penetrating into running buffer. Good linear detection response between concentration of S protein (**c**) and SARS-CoV-2 (**d**) with respect to the change in differential GH shift obtained by optimal configuration: 32 nm Au thin film/2-QL Bi_2_Se_3_/3-layer graphene. Solid curve stands for extracted linear response interval, and dotted curve demonstrates a linear fitting. Abbreviation: Au (42 nm)_ Bi_2_Se_3_ (2 QL)_graphene (3L) in Figure 6a denotes the optimal plasmonic configuration: 32 nm Au film−2-QL Bi_2_Se_3_-3-layer graphene.

To quantitatively monitor the concentrations of target analytes, it is necessary to determine a linear response interval for our optimal plasmonic configuration: 32 nm Au film−2-QL Bi_2_Se_3_-3-layer graphene. Figure S2 showed a linear response of GH shift for a tiny RI variation as small as 10^−6^ RIU. In contrast to 32 nm Au thin film, 3-layer graphene coated on 32 nm Au film, the optimal configuration: 32 nm Au film−2-QL Bi*2*Se_3_-3-layer graphene can provide an enhancement factor (EF) of 2 × 10^5^. It was assumed that significantly local field intensity enhancement should contribute to obtain such a high EF. It is well-known that typical nanoscale heterogeneous configurations, such as C-shaped [40], Φ-shaped [41], bowtie-shaped [42] and ring-shaped [43] nanostructures, have shown significant field enhancement. To verify the proposal for producing such a high EF, the electric field distribution of 32 nm Au film−2-QL Bi_2_Se_3_-3-layer graphene was further simulated via finite element analysis method, as shown in Figure 6a,b. It clearly showed that, under the illumination of 632.8 nm, there was a significantly enhanced electric field close to the Bi_2_Se_3_/graphene sensing interface. Moreover, the electric field intensity decayed exponentially into a running buffer, resulting in a penetration depth of 150 nm. In addition, the charge-transfer mechanism generated by difference in work function (Fermi level) was proposed to contribute to giant electric field enhancement. It has been reported that the work function of Au film is ~5.54 eV and the work function of 2-QL Bi_2_Se_3_-3-layer graphene is ~4.75 eV [22]. The large difference in work function can induce strong band bending, eventually resulting in strong charge-transfer. Thus, the direct-write orientation of charge transfer in optimal configuration would be from Bi_2_Se_3_/Graphene heterostructures to Au thin film.

Employing the linear response interval of 32 nm Au film-2-QL Bi_2_Se_3_-3-layer graphene, the feasibility of monitoring SARS-CoV-2 and its S protein was theoretically studied. It was predicted that the molecular weight (MW) of SARS-CoV-2 is approximately 800 kDa [44], which is much larger than that of S protein 180 kDa [45]. According to Equation (2), linear relationships between change in differential GH shift (µm) and detection concentration (nM) of adsorbed analytes were plotted, as shown in Figure 6c,d. For a minor RI variation of 0.000002 RIU, a good linear detection interval (Figure 6c) for S protein is from 0 to 59.74 nM, which is described by an equation Δ*GHS_d_* = 30.1047 *c_Sprotein_. Here, the *c_Sprotein_* denotes the concentration of S protein solution. More importantly, our proposed biosensor also exhibited an excellent response interval (0–13.44 nM) for quantitatively monitoring SARS-CoV−2. The linear response can be expressed by such an equation Δ*GHS_d_* = 133.7987 *c_SARS-CoV−2_, where *c_SARS-CoV−2_* stands for the concentration of SARS-CoV−2 solution. Therefore, our proposed plasmonic biosensor has shown great feasibility in quantitatively monitoring SARS-CoV-2 and its S protein.

## 4. Conclusions

In this work, we theoretically proposed a sensitivity enhanced SPR biosensor by incorporating Bi_2_Se_3_/graphene heterostructures with conventional plasmonic Au film. The biosensing performance of our proposed configuration was designed by GH shift, superior to phase modulation. The energy loss could approach zero by introducing the Bi_2_Se_3_/graphene heterostructures. The best configuration employed to achieve the highest photon absorption is 32 nm Au film/2-QL Bi_2_Se_3_/3-layer graphene, producing an ultralow (approaching zero) reflectivity of 5.4402 × 10^−9^ and a highest GH shift of −1.0202 × 10^4^ µm. Responding to a small RI variation in 0.0012 RIU, the proposed biosensor was able to provide an ultra-high sensitivity of 8.5017 × 10^6^ µm/RIU. To extend the potential application in biosensing, our proposed biosensor was employed to theoretically predict the adsorption response of SARS-CoV−2 in a tiny RI variation as low as 0.000002 RIU. For SARS-CoV−2, a linear relationship (0~13.44 nM) was determined by an equation Δ*GHS_d_* = 133.7987 *c_SARS-CoV−2_. For its S protein, another linear relationship was described by an equation Δ*GHS_d_* = 30.1047 *c_S protein_ and there was a linear detection interval (0~59.74 nM). In view of these observations, we concluded that, our optimal plasmonic biosensor shows great promise in quantitatively monitoring SARS-CoV−2 and other infectious viruses for practical applications.

## Figures and Tables

**Table 1:** Comparison of GH shift-based SPR sensors under the excitation of 632.8 nm.

2D Material Configuration	Sensitivity (µm/RIU)	Change in RI	Ref.
Au/ITO/MoSe_2_/graphene	5.075 × 10^5^	0.002	[32]
Au/MoS_2_/graphene	3.509 × 10^5^	0.002	[31]
Au/graphene/PtSe_2_	1.37 × 10^5^	0.005	[39]
Au/Bi_2_Se_3_/graphene	8.5017 × 10^6^	0.0012	Current work

## Data Availability

The data presented in this study are available upon request from the corresponding author (yufengyuan@dgut.edu.cn).

## Supplementary Materials

The supplementary materials are available online at https://www.mdpi.com/article/10.3390/nano12224078/s1. Supplementary figures: Figure S1: Change in resonance angle (a) and obtained angle sensitivity (b) by varying the number of Bi2Se3 QLs (0–5) and graphene (0–5) for a defined RI variation of 0.0012 RIU. Figure S2: Comparison of SPR sensing performances generated by 32 nm Au thin film, 3-layer graphene coated on 32 nm Au thin film and 32 nm Au film deposited with 2-QLs Bi2Se3 and 3-layer graphene for a tiny RI variation (as low as 10^−6^ RIU).
