# Supplementary material for: Sensitivity Enhanced Plasmonic Biosensor Using Bi2Se3-Graphene Heterostructures: A Theoretical Analysis
Source: Nanomaterials (Basel). 2022 Nov 19;12(22):4078. doi: 10.3390/nano12224078 (PMC9696186; doi:10.3390/nano12224078)
Supplement: Supplementary file 1 [file nanomaterials-12-04078-s001.zip › nanomaterials-2030799-supplementary.pdf]

Supplementary Materials

Sensitivity Enhanced Plasmonic Biosensor Using Bi_2Se_3-Graphene Heterostructures: A Theoretical Analysis

Fusheng Du [1,2], Kai Zheng [3,4], Shuwen Zeng [5] and Yufeng Yuan [1,3,*]

[1] School of Electronic Engineering and Intelligentization, Dongguan University of Technology, Dongguan 523808, China
[2] School of Information and Optoelectronic Science and Engineering, South China Normal University, Guangzhou 510631, China
[3] Shenzhen Key Laboratory of Photonics and Biophotonics, Key Laboratory of Optoelectronic Devices and Systems of Ministry of Education and Guangdong Province, College of Physics and Optoelectronic Engineering, Shenzhen University, Shenzhen 518060, China
[4] School of Civil Aviation, Northwestern Polytechnical University, Xi'an 710072, China
[5] Light, Nanomaterials & Nanotechnologies (L2n), CNRS-ERL 7004, Université de Technologie de Troyes, 10000 Troyes, France
* Correspondence: yufengyuan@dgut.edu.cn

Supplementary Figures

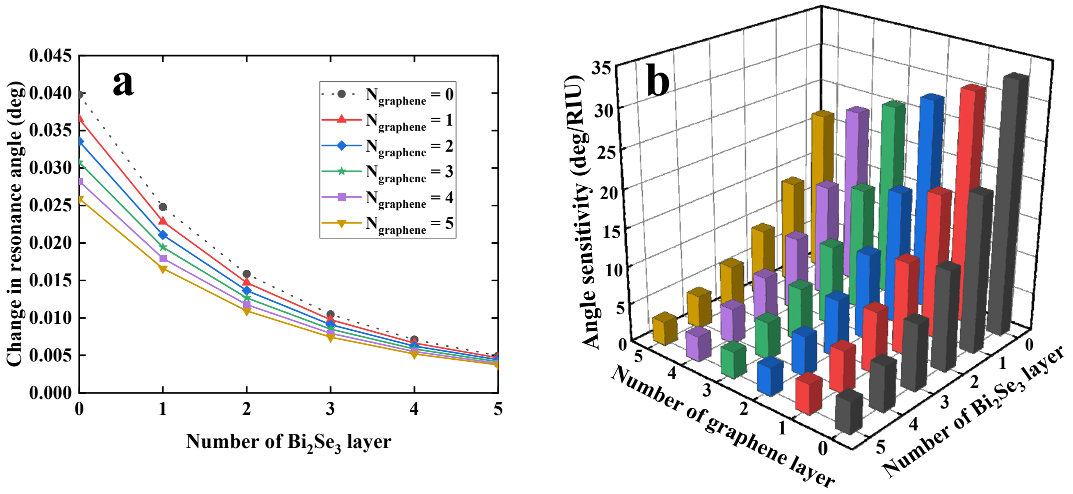

Figure S1 Change in resonance angle (**a**) and obtained angle sensitivity (**b**) by varying the number of Bi_2Se_3 QLs (0-5) and graphene (0-5) for a defined RI variation of 0.0012 RIU.

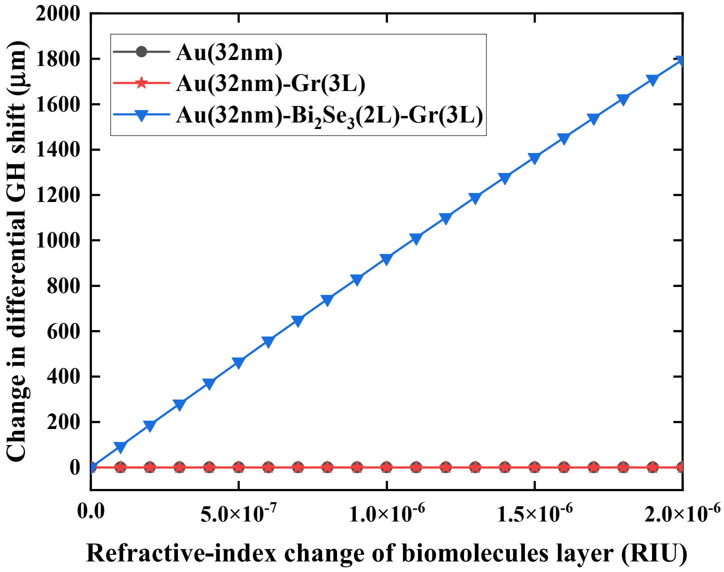

Figure S2 Comparison of SPR sensing performances generated by 32nm Au thin film, 3-layer graphene coated on 32 nm Au thin film, and 32 nm Au film deposited with two-QLs Bi_2Se_3 and three-layer graphene for a tiny RI variation (as low as 10^{-6} RIU).
